# Enhancement of the therapeutic efficacy of mesenchymal stem cell-derived exosomes in osteoarthritis

**DOI:** 10.1186/s11658-023-00485-2

**Published:** 2023-09-28

**Authors:** Zehao Zhang, Sheng Zhao, Zhaofeng Sun, Chuanxing Zhai, Jiang Xia, Caining Wen, Yuge Zhang, Yuanmin Zhang

**Affiliations:** 1 School of Clinical Medicine, Jining Medical University, Jining, 272067 Shandong China; 2 Department of Chemistry, The Chinese University of Hong Kong, Shatin, Hong Kong, SAR China; 3 Department of Joint Surgery and Sports Medicine, Affiliated Hospital of Jining Medical University, Jining Medical University, Jining, 272029 Shandong China

**Keywords:** Osteoarthritis, Mesenchymal stem cells, Exosomes, Bioengineering

## Abstract

Osteoarthritis (OA), a common joint disorder with articular cartilage degradation as the main pathological change, is the major source of pain and disability worldwide. Despite current treatments, the overall treatment outcome is unsatisfactory. Thus, patients with severe OA often require joint replacement surgery. In recent years, mesenchymal stem cells (MSCs) have emerged as a promising therapeutic option for preclinical and clinical palliation of OA. MSC-derived exosomes (MSC-Exos) carrying bioactive molecules of the parental cells, including non-coding RNAs (ncRNAs) and proteins, have demonstrated a significant impact on the modulation of various physiological behaviors of cells in the joint cavity, making them promising candidates for cell-free therapy for OA. This review provides a comprehensive overview of the biosynthesis and composition of MSC-Exos and their mechanisms of action in OA. We also discussed the potential of MSC-Exos as a therapeutic tool for modulating intercellular communication in OA. Additionally, we explored bioengineering approaches to enhance MSC-Exos’ therapeutic potential, which may help to overcome challenges and achieve clinically meaningful OA therapies.

## Development and characterization of osteoarthritis

Osteoarthritis (OA) is a progressively aggravating disease that can affect both small joints (such as hand joints) and large joints (such as knee and hip joints). Clinical symptoms of OA include knee pain, restricted movement, inflammation of the synovium, joint deformity, and even disability [1]. In fact, these clinical manifestations reflect various phenotypes of OA, including chronic pain phenotype, inflammatory phenotype, mechanical overload phenotype, minimal arthropathy phenotype, etc. [2]. The prevalence of OA is increasing year-over-year, with over 500 million people affected worldwide [3], and it ranks as the 11th most disabling disease in the world. According to statistics, about 9.6% of men and 18% of women over the age of 60 worldwide have symptomatic OA. Understanding the underlying mechanisms of OA will help to develop new treatments for future clinical needs.

**Fig. 1:**
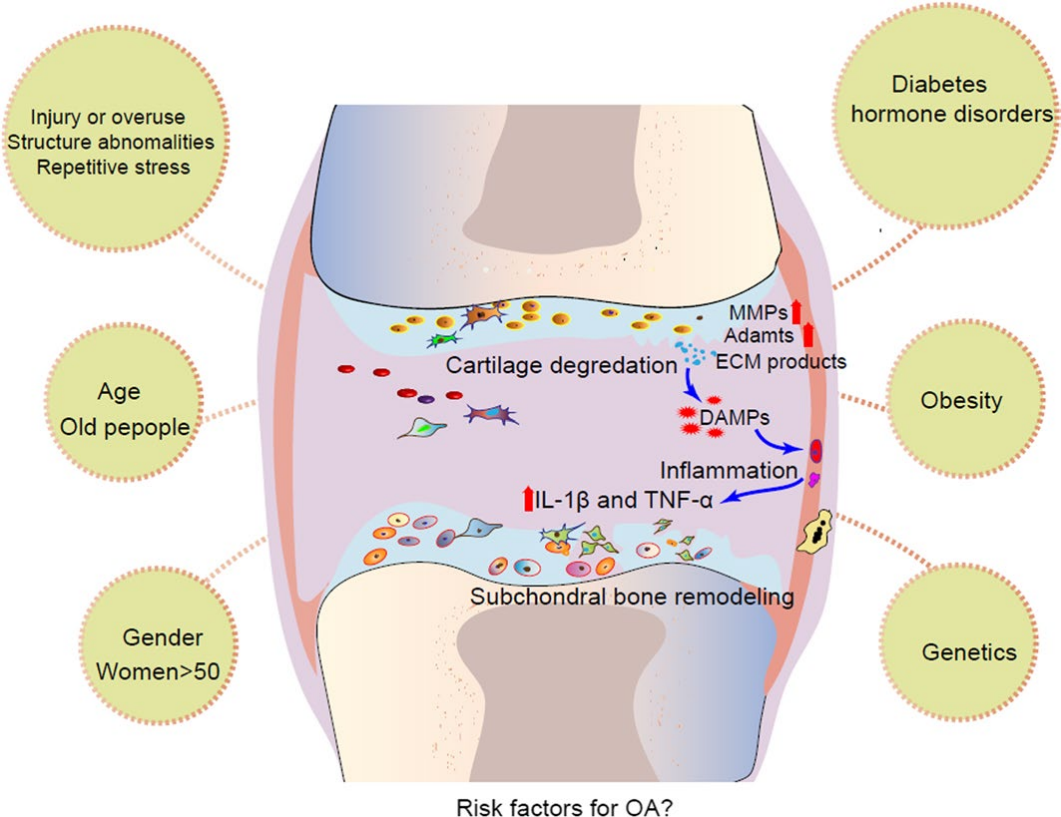
Risk factors and pathological mechanisms of OA. Specific systemic risk factors (age, obesity, and sex) and mechanical factors (joint injury or overuse) can cause OA-like cartilage damage. Changes in the cartilage microenvironment, such as cellular secretion of matrix-degrading enzymes, reactive oxygen species, and cytokines, can accelerate the process of cartilage degeneration, resulting in the loss of the chondrocyte phenotype. The degradation products of cartilage and extracellular matrix (ECM) components can also be released into the joint cavity as damage-associated molecular patterns (DAMP) will boost the synovial cells and macrophages to secrete different types of inflammatory mediators. In addition, remodeling of the subchondral plate and calcified cartilage also reflects changes in the mechanical loading of cartilage and subchondral bone

While OA was once thought to be characterized primarily by the destruction of articular cartilage, recent evidence suggests that OA is a disease with whole joint damage and dysfunction [4]. During the progression of OA, pathological changes in the joint include cartilage damage, subchondral bone remodeling, inflammatory activation of the synovial membrane, and degeneration of periarticular tissues (connective and muscular tissues) and soft tissues (ligaments, tendons, and menisci) [5, 6]. A variety of factors contribute to pathological changes in OA joints [7], including aging, inflammation, trauma, oxidative stress, mechanical loading, and genetic and metabolic disorders (Fig. 1) [8]. The pathogenesis of OA involves various molecular pathways, such as protein hydrolysis/catabolic enzymes [e.g., matrix metalloproteinases (MMP), collagenases, and aggregated protein kinases], oxidation (superoxide and hydrogen peroxide), nitrosylation (nitric oxide and peroxynitrite) stress, inflammatory cytokines [interleukin-1β (IL-1β) and tumor necrosis factor-α (TNF-α)], PGE2, cAMP, and many others, including transforming growth factor-β (TGF-β), c-Jun N-terminal kinase (JNK), and p38. Studies have revealed that certain miRNAs are associated with the development of OA [9], while other miRNAs are associated with the inhibition of OA [10–13]. Additionally, chondrocyte apoptosis plays an important role in the degeneration of OA articular cartilage [14, 15]. Subchondral bone, which is the cortical bone and trabecular bone structure beneath the articular cartilage, also plays an important role in the pathogenesis of OA, as it may contribute to cartilage degeneration through mechanical alterations or paracrine-mediated bone–chondral interactions [16]. Cytokines from inflammatory synovial fibroblasts (SFB) and infrapatellar fat pads (IPFP) may lead to the release of various proinflammatory mediators, which not only extensively alter synovial tissue structure and function, but also promote cartilage matrix damage and accelerate the development of OA [17, 18].

## Landscape of MSCs-mediated therapies in osteoarthritis

**Fig. 2:**
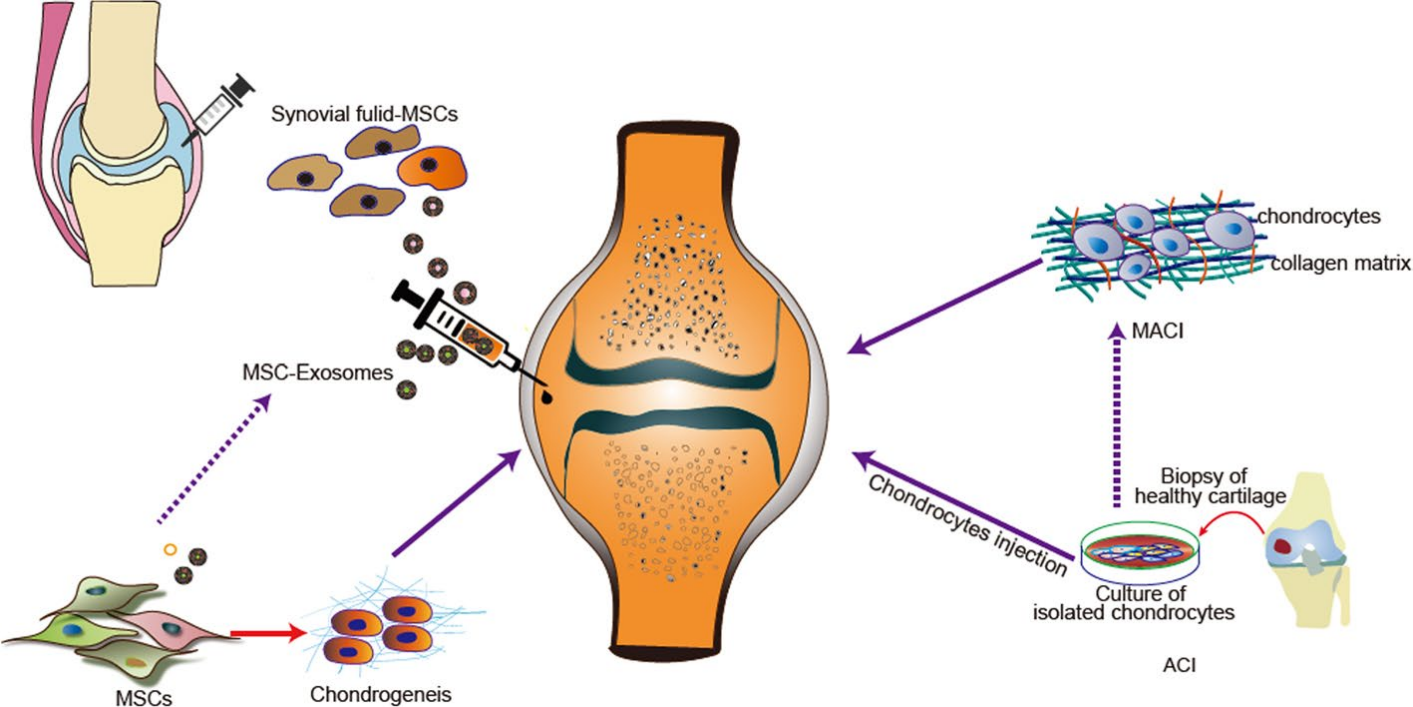
Current cell-based and cell-free therapies for osteoarthritis. Cell-based treatments for OA rely on regenerating injured cartilage while reducing ongoing inflammation in the affected joint. Autologous cultured chondrocytes based on ACI and MACI have been shown to be useful for cartilage tissue repair. MSCs are emerging as powerful self-renewal agents for osteoarthritis treatment. In addition, mesenchymal stem cell-derived exosomes (MSC-Exos) could replace MSCs and represent a novel cell-free therapy for OA

Current treatment options for OA have limitations. Traditional medical treatments only control symptoms and delay injury but cannot reverse the progression of OA. Thus end-stage degenerative joint diseases may require artificial joint replacement and osteotomy, but these procedures can result in serious complications such as postoperative stiffness, pain, thromboembolism, and prosthetic infection. The 10-year postsurgery risk of failure of total knee arthroplasty requiring revision surgery is 5% [19]. The rapid growth of cell transplantation and tissue regeneration methods for various diseases over the past few decades provides new avenues future for OA treatment. Figure 2 illustrates several of the most widely used cell transplant or cell-derived bioactive cargoes for cartilage repair. However, some methods, such as autologous chondrocyte implantation (ACI) or matrix-induced autologous chondrocyte implantation (MACI), have not been widely utilized for reasons such as the formation of fibrocartilage [20]. In contrast, MSCs have shown promise as ideal seed cells for the effective treatment of OA without the aforementioned drawbacks. MSCs are self-renewable pluripotent stem progenitor cells that can differentiate into various cell lines, including osteoblasts, adipocytes, and chondrocytes [21] [22]. MSCs can be induced to differentiate into chondrocytes and form cartilage matrix in a targeted manner, and they exhibit high therapeutic efficacy and safety due to their unique immunological properties. Allogeneic MSCs even have advantages over autologous bone marrow-derived MSCs (BM-MSCs) in clinical outcomes in elderly patients with reduced cartilage degeneration [23]. More specially, studies have found a high content of synovial fluid-derived MSCs (SF-MSCs) in the joint fluid of patients with OA [24]. Compared with the commonly used BM-MSCs, SF-MSCs have several advantages. They can be derived from medical wastes such as patients’ arthroscopic irrigation fluid and possess significantly higher chondrogenic differentiation capacity [25–27]. Furthermore, intraarticular injections of SF-MSCs have shown chondroprotection in an animal model of OA or articular cartilage defects [28–31]. Adipose tissue-derived MSCs (AD-MSCs) are another stem cell option for the treatment of OA. Adipose tissue has the advantages of being a wide source and having a better safety profile. Comparing the efficacy and safety of BM-MSCs and AD-MSCs in knee OA, AD-MSCs were shown to be more effective [32]. In addition, autologous sources such as endometrium [33] and peripheral blood (PB) [34], and allogenic sources such as placenta [35], umbilical cord [36], and amniotic fluid-derived MSCs [37] are accessible source of stem cells for OA treatment. We summarized the role of MSCs in the treatment of OA as shown in Table1.

**Table 1:** Clinical trials of MSCs transplantation for knee OA

MSC source	Cell population	Follow-up	Outcomes	Trial registration number	Reference
BM-MSCs	100 × 10^6^	12 months	Clinical symptom improvement	NCT02365142 (phase II)	[40]
Allogenic placenta-derived MSCs	0.5–0.6 × 10^8^	24 weeks	Improve symptoms of knee osteoarthritis	IRCT2015101823298N	[35]
BM-MSCs	8–9 × 10^6^	12 months	Pain alleviation and improved walking abilities	NCT00550524	[41]
BM-MSCs	40 × 10^6^	6 months	Significantly improved pain scores	NCT01504464 (phase I/II)	[42]
Infrapatellar fat pad-derived MSCs	1.89 × 10^6^	3 months	Improved joint function, pain level	–	[43]
BM-MSCs	10 or 100 × 10^6^	12 months	Improvement in pain and symptoms	NCT02123368 (phase I/II)	[44]
Umbilical cord-derived (UC) MSCs	20 × 10^6^	12 months	Alleviate symptoms of knee joint pain	NCT02580695 (phase I/II)	[45]
BM-MSCs	40.9 × 10^6^	12 months	Pain relief, cartilage regeneration	NCT01183728 (phase I/II)	[46]

Apparently, MSCs from multiple sources have successfully moved toward the clinical trial stage for the treatment of OA. According to the follow-up results, MSCs showed positive results in the treatment of knee OA. In addition, these clinical trials have somewhat illustrated the biological safety of MSCs. However, while we vigorously promote MSCs toward the clinic to serve patients, we should also be aware of some of their possible risks. For example, the follow-up period of the current study is not long enough to predict whether MSCs will pose long-term biosafety problems. To obtain a sufficient amount of MSCs, patients may need to undergo multiple invasive procedures. And the renewal and differentiation capacity of MSCs declines due to the amplification process [38, 39].Therefore, there is an urgent need to develop effective agents that have the efficacy of MSCs as seed cells, while avoiding their potential risks, and could deliver relevant drugs for the treatment of OA cartilage degeneration.

Fundamental studies have shown that ability of MSCs to direct differentiation and paracrine secretion is the key factor to their therapeutic benefit in OA [47]. Direct differentiation into chondrocytes can play a reparative role by homing MSCs to the site of cartilage defects and proliferating and differentiating into functional cartilage or cartilage-like tissue. The process involves the recognition of chemokine ligands in the bone and joint fluid and binding to chemokine receptors (CXCR) on MSCs [48], thereby allowing MSCs to migrate to the damaged tissues [49]. Common chemokines such as the stromal derived factor-1 (SDF-1)/CXC chemokine receptor 4 (CXCR4) axis, osteopontin (OPN) has been used to help this “homing” process [50]. Of course, localized inflammation, degenerative lesions, and mechanical factors have also been shown to promote the homing of these stem cells to a damaged tissue [51]. As mentioned previously, MSCs have the ability to differentiate into chondrocytes. Since the natural regenerative capacity of cartilage is limited locally in OA, stem cell therapy is of great significance for cartilage regeneration in OA. The targeted differentiation of MSCs into chondrocytes is dependent on the local environment and signaling pathways. Cell–matrix interaction is a key factor influencing the biological response of MSCs, which are stimulated by the cartilage matrix to differentiate into the chondrocyte lineage after homing to the site of cartilage defects [52]. And in the OA microenvironment, transcription factors Sox9 and Runx2 and bone morphogenetic protein (BMP) signaling pathway regulate chondrogenesis [53] [54]. Apart from the fact that the differentiation of MSCs into chondrocytes is central to tissue repair, the paracrine mechanisms, however, are considered more important for the therapeutic effects of MSCs in OA. MSCs regulate local inflammation, apoptosis, and cell proliferation by secreting soluble factors and extracellular vesicles into the surrounding environment [55]. In summary, the mechanisms by which MSCs act on OA mainly rely on [1] homing to the site of cartilage defects, [2] chondrogenic differentiation to repair cartilage defects, and [3] paracrine secretion of tissue repair factors. Among the factors secreted by MSCs, the exosomes are involved in many physiological and pathological processes of OA. In recent years, the role and therapeutic potential of MSC-Exos in OA has received increasing attention.

In this review, we summarized the current state of research and major clinical challenges of using MSC-Exos in the treatment of OA and discussed bioengineering solutions to enhance MSC-Exos for the treatment of OA, with the hope of providing an important reference for developing more effective and predictable methods of using MSC-Exos for OA treatment.

## Biogenesis and characterization of MSCs-derived exosomes

**Fig. 3:**
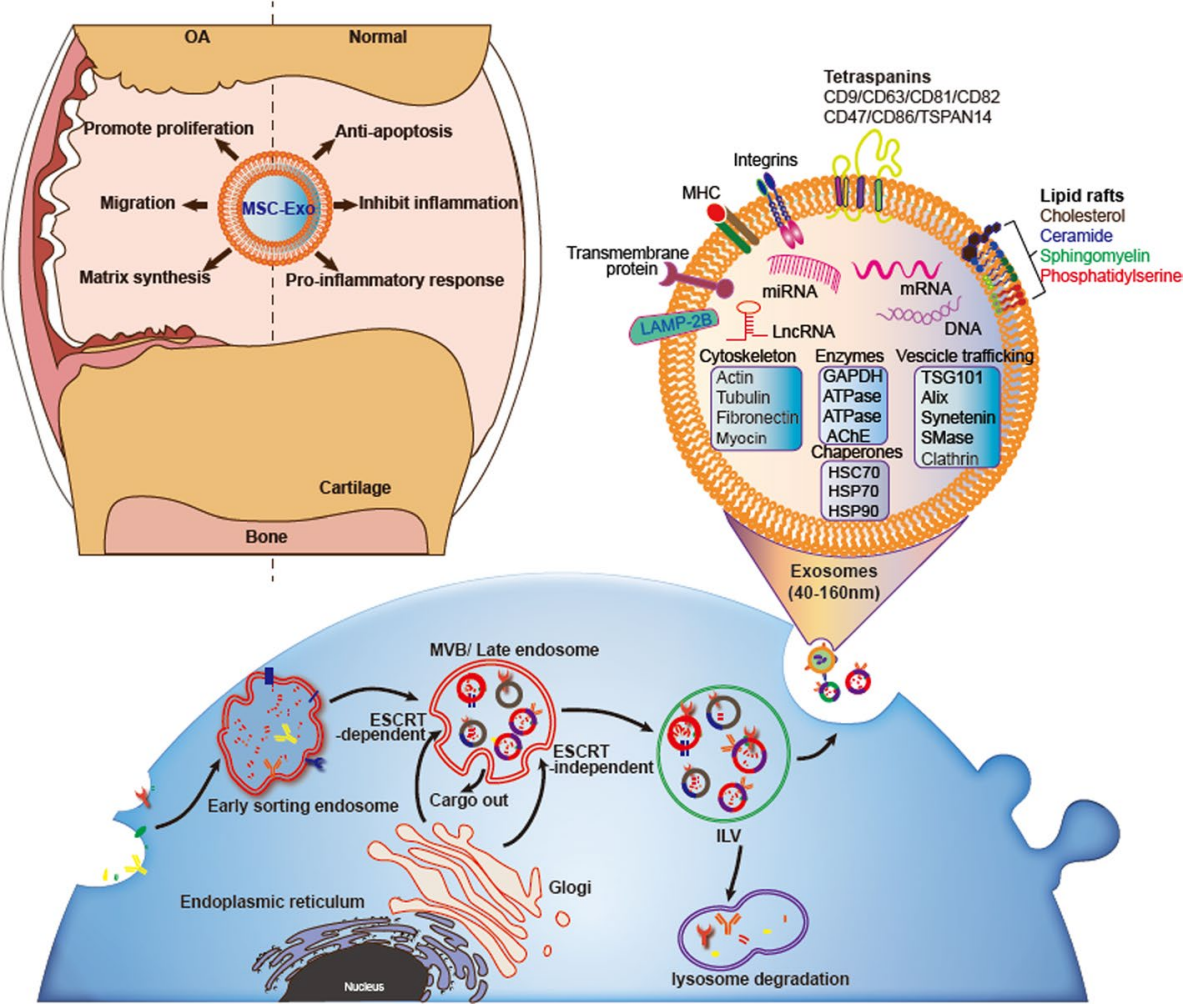
MSC-Exos: generation, structural composition, and protective effects on joint cartilage. MSC-Exos are nanoscale extracellular vesicles that originate from the inward budding of late endosomal membranes called MVBs and are released after fusion with the plasma membrane. The major components of MSC-released exosomes are lipid membranes rich in cholesterol, sphingomyelin, ceramide, and lipid raft proteins, which protect the contents from degradation or fusion with the membranes of target cells. In addition, exosomes of MSCs carry small nucleic acid molecules that play an important role in cartilage tissue regeneration, cartilage immunomodulation, and other processes

Exosomes are nanoscale extracellular vesicles with a diameter of 50–150 nm [56] and enclosed by a lipid bilayer. They originate from the endocytic pathway where endosomal membranes bud inward to the lumen to form intraluminal vesicles (ILVs). As early endosomes mature into late endosomal multivesicular bodies (MVBs), MVBs fuse with the plasma membrane to release ILVs/exosomes (Fig. 3) [57]. Exosomes undergo a series of regulatory processes that affect both the formation of exosome structure and cargo sorting. The endosomal sorting complex required for transport (ESCRT) pathway is the first pathway identified for exosome biogenesis. It consists of four protein complexes, namely ESCRT-0, ESCRT-I, ESCRT-II, and ESCRT-III, as well as the proteins ALG-2-interacting protein X (ALIX), VTA1, and vacuolar protein sorting-associated protein 4 (VPS4). These ESCRT components assemble on the MVB membrane to regulate the assembly of cargoes and the formation of ILVs, promoting the formation and secretion of exosomes [58, 59]. In the endosomes, ESCRT-0, ESCRT-I, and ESCRT-II are responsible for recognizing ubiquitinated proteins and loading them into the lumen of the endosome [60]; ubiquitinated membrane proteins are recognized and incorporated into the MVB to participate in ILV formation, and non-ubiquitinated proteins are incorporated into the plasma membrane or Golgi complex [61]. Specifically, ESCRT-0 is involved in cargo aggregation and ubiquitination of endocytic receptors [62, 63], while ESCRT-I forms a complex with ubiquitinated proteins that activate ESCRT-II. Together, ESCRT-I and ESCRT-II complexes drive endosomal membrane bending to form ILVs [62, 63]. ESCRT-III is activated by ESCRT-II to recruit auxiliary proteins such as ALIX and VPS4, which together assist ILV formation in the MVB [59, 60]. Overall, the interplay among various ESCRT components allows cargo to be sequestered in ILVs and eventually excreted extracellularly to form exosomes. For ESCRT-independent pathways of exosome biogenesis, the neutral sphingomyelinase 2 (nSmase2) contributes to ceramide and sphingomyelinase hydrolysis, inducing MVBs to bud inward, and ceramide can cause the MVB membrane to spontaneously bend to form ILVs and eventually fuse with the cytosol to release exosomes [64]. However, tetraspanin proteins CD63 and CD81 also sort the associated proteins and ligands to ILVs [65, 66]. Other RAS-associated proteins Rab27A or Rab27B are indispensable to producing plasma membrane exosomes. Deletion of Rab27A or Rab27B alters the morphology of the MVB and affects its docking with the plasma membrane [67]. Various substances that are involved in the regulation of exosome biogenesis, such as ESCRT pathway-related components (TSG101 and ALIX), tetrapeptides (CD63, CD81, and CD9), membrane transport proteins (Rab proteins), and lipid components (ceramides), eventually become part of exosomes, conferring corresponding ligand effects, or becoming markers for identifying exosomes [68, 69].

Exosomes are enriched not only in protein components but also in various types of nucleic acids, including mRNA, microRNA (miRNA), ribosomal RNA, long non-coding RNA (lncRNA), and DNA [70]. Despite accumulating evidence of the biological effects of exosomal nucleic acids, the loading mechanisms of these cargoes remain to be elucidated. Existing studies suggest that RNA encapsulation is closely related to RNA binding proteins (RBPs), with most RNAs being transferred from the nucleus to specific cellular sites associated with RBPs at the exosome biogenesis sites. RBPs serve as a bridge between RNA and exosome biogenesis [71, 72]. Typical RBPs such as heterogeneous nuclear ribonucleoprotein A2/B1 (hnRNPA2B1) are associated with exosome miRNA sorting [73], while studies have shown that miRNAs may be regulated by posttranscriptional modifications upon entry into exosomes [74]. In addition, RBPs regulate the enrichment of ncRNAs, short mRNAs, and mRNA fragments into exosomes [75]. Despite the knowledge that RBPs are important for RNA entry into exosomes, the mechanisms, such as the role of RBPs, specific RNA sequence motifs, and differential affinity for membrane lipids, still need to be elucidated in depth [76, 77]. However, there is an ambiguity in studies on DNA loading into exosomes, which is currently thought to be related to aging and DNA damage [78–80].

Enriched with various factors, exosomes become a means of exchanging molecular information for intercellular communication. Exosomes interact with receptor cells through their surface proteins and can fuse with the plasma membrane of receptor cells to release their contents, thus triggering signal transduction and ultimately affecting the function of the receptor cells [81]. MSC-Exos can be produced under both pathological and physiological conditions, and these exosomes can function similarly to MSCs themselves [82]. Recent studies have found that MSC-Exos can penetrate cartilage tissue as nanoscale transport vehicles and overcome tissue barriers for drug delivery into the dense articular cartilage matrix, which may provide a new means of treating cartilage tissue defects. We present a summary of studies on the role of extracellular vesicles in osteoarthritis in Table 2. By summarizing the previous reports, the potential of MSC-Exos for the treatment of OA in vitro and in vivo showed great promise. Studies on the use of MSC-Exos to delay OA progression or reverse progression toward OA have shown promising results. MSC-Exos can regulate chondrocyte proliferation, apoptosis, extracellular matrix degradation, and cartilage inflammation (Fig. 3) [83]. These studies observed that the MSC-Exos promote cartilage repair through a multifaceted mechanism involved in chondrocyte proliferation (MSC-Exos dose-dependent proliferation), increased infiltrative migration (migration of chondrocytes into the injured tissue), enhanced matrix synthesis (increase in type II collagen), attenuated apoptosis (increase in the anti-apoptotic gene BCL-2), inflammatory suppression (reduction of IL-1β and TNF-α), and immunomodulation (increase in M2 macrophages) [84]. A growing number of studies have confirmed the potential of MSC-Exos for the treatment of OA. Of course, the function of MSC-Exos as an important signaling mediator for intercellular communication involves complex contents and signaling pathways. We cover the discussion of related contents and mechanisms in the following table.

**Table 2:** Summary of the role of MSC-Exos role in osteoarthritis therapies

Exos source	Cargo	Molecular mechanism	Action effect	Reference
BM-MSC-Exos	Curcumin	In vivo and ex vivo: regulate miR-124/ NF-κB and miR-143/ROCK1/TLR9	Reduce chondrocytes apoptosis	[85]
Human MSC-Exos	lncRNA KLF3-AS1	In vivo and ex vivo: target lncRNA-KLF3-AS1/miR-206/GIT1 axis and promote proliferation	Promote proliferation and reduce apoptosis in carilage repaire	[86]
Dental pulp stem cell-derived Exos	–	In vivo: downregulate TRPV4 to suppress osteoclastogenesis	Protect cartilage degradation, ameliorate inflammatory responses	[87]
BM-MSC-Exos	lncRNA MEG-3	In vivo: attenuate excess cartilage degradation and subchondral bone remodeling Ex vivo: enhance the synthesis of type II collagen and inhibition of IL-1β-induced senescence and apoptosis	Enhance cartilage-protective effects	[88]
BM-MSC-Exos	–	In vivo: low-intensity pulsed ultrasound (LIPUS)-mediated BM-MSC-Exos to inhibit IL-1β-induced activation of NF-κB pathway	Antiinflammatory effects, promote cartilage regeneration	[89]
BM-MSC-Exos	miR-92a-3p	In vivo and ex vivo: miR-92a-3p directly targeting 3'-UTR and inhibit WNT5A expression	Regulation of cartilage homeostasis and chondrogenesis	[90]
Human embryonic stem cell-induced MSC-Exos	—	In vivo: increase synthesis of type II collagen and decreased expression of of ADAMTS5	Remodel the synthesis and degradation of extracellular matrix	[91]
Infrapatellar fat pad MSC-Exos	miR-100-5p	In vivo and ex vivo: miR-100-5p-mediated inhibition of mTOR autophagic pathway,	suppression of apoptosis and homeostasis of metabolic processes	[92]
SF-MSC-Exos	Kartogenin (KGN)	In vivo and ex vivo: effectively induce differentiation of SF-MSCs toward chondrocytes	Promote cartilage development	[28]
Human embryonic stem cell-derived MSC-Exos	–	In vivo: enhance s-GAG synthesis, inhibited IL-1β-induced nitric oxide and MMP13 production,	regulate cartilage homeostasis, reduce inflammation, and enhance chondrocytes proliferation	[93]

## Exosomes/ncRNAs as mediating MSC-free based therapy of osteoarthritis

As previously discussed, exosomes can biogenetically sort various physiologically active substances, such as miRNA, mRNA, and proteins, which can be delivered to target cells and lead to significant changes in function and phenotype [20, 94]. These bioactive cargoes play a key role in intercellular communications during OA development [51]. Among these cargoes, exosome-miRNAs have been extensively studied in recent years. MiRNAs inhibit mRNA translation and splicing by binding to the non-coding regions of target mRNAs, which affect downstream pathways by blocking target gene expression [95–97]. Approximately 150 miRNAs are enriched in exosomes, and multiple miRNAs have key therapeutic benefits in OA [98]. Table 3 lists the most widely explored MSC-Exos miRNAs involved in OA. As shown in Table 3, a large number of studies have demonstrated the critical role of MSC-Exo-derived miRNAs in OA. By analyzing the targets of miRNAs, researchers identified multiple signaling pathways associated with OA. MiRNAs, as a small fraction of the contents in MSC-Exos, exhibit powerful biological efficacy. The signaling pathways of different miRNAs were synergizing to exert beneficial therapeutic effects on OA.

**Table 3:** MiRNAs known to involve in MSC-derived exosome-based osteoarthritis therapies

miRNAs	Target	Function	Reference
miR-22	PPAR-α, BMP-7	Protect cartilage degradation by suppressing PPAR-α and BMP-7 expression, thus significantly decreasing MMP-13 expression	[104]
miR-23	PKA	Enhance chondrogenesis via PKA signaling	[105]
miR-92a	Noggin3	Enhance chondrocyte proliferation and ECM matrix synthesis via targeting noggin3 to activate PI3K/AKT/mTOR pathway and suppress cartilage degradation	[106]
miR-92a-3p	WNT5A	Induce cartilage tissue regeneration and prevent osteoarthritis	[90]
miR-95-5p	HDAC2/8	Promote cartilage regeneration and attenuate OA progression	[107]
miR-125b and miR-320	ADAMTS-4 (aggrecanase-1) and MMP-13	Prevent extracellular matrix degradation via downregulating the expression of ADAMTS-4 (aggrecanase-1) and MMP-13	[108, 109]
miR-140	RALA	Target RALA and stimulate ECM production by increasing SOX9 expression, which further promotes the production of cartilage matrix components by downregulating RALA	[110]
miR-140-5p	Wnt and YAP	Enhance proliferation and migration of articular chondrocytes	[111]
miR-320c	MMP13	Promote chondrocyte proliferation and cartilage homeostasis	[112]

Besides miRNAs, lncRNAs have also shown therapeutic effects on OA. LncRNAs act as regulatory molecules by binding miRNA to mRNA binding sites, thereby reducing the expression of miRNA or mRNA [99]. Liu et al. found that overexpression of lncRNA-KLF3-AS1 in MSC-Exos effectively ameliorated IL-1β-induced chondrocyte injury. The lncRNA exerted a role of competing endogenous RNAs to suppress miR-206 expression, ultimately promoting cartilage repair [100]. Additionally, exosome-lncRNA MEG3 alleviates IL-1β-induced chondrocyte senescence and apoptosis, which explains its beneficial therapeutic effects on OA via BM- s [88]. Recent studies have shown that exosomal circRNAs represent a promising molecular sponge, competing for the regulation of miRNA expression, thus affecting a variety of diseases, including OA [101]. For example, exosomal circ-BRWD1 serves as a miR-1277 sponge to reduce chondrocyte viability and promote apoptosis, inflammation, and ECM degeneration via regulating TRAF6. This may provide a promising novel strategy for OA treatment [102]. Similarly, exosome-mediated knockdown of circ_0001846 regulates chondrocyte injury through a novel exosome/miR-149-5p/WNT5B pathway [103]. Understanding the important regulatory role of exosomal ncRNAs in osteoarthritis will provide novel therapeutic options for future clinical applications.

## Immunoregulatory potential and antiinflammatory effects of MSC-Exos on OA

Synovial inflammation is a key feature of OA and involves various types of immune cells, including macrophages, T cells, and B cells, and their secreted inflammatory factors within the synovial fluid of affected patients [5]. Numerous studies have established the immunomodulatory potential of MSCs in inflammatory diseases such as OA [113–115]. As a paracellular secretory product, MSC-Exos are a safer and more effective means of regulating inflammation and immunity in the joint cavity of patients with OA. Previous studies have shown promising results that MSC-Exos exert anti-OA effects by regulating the biological behavior of macrophages. Shen et al. reported that the highly-expressed C–C chemokine receptor type 2 in exosome can bind to proinflammatory chemokine CCL2 and inhibit its activity, thus effectively preventing macrophage accumulation and inflammation [116]. In another study, the researchers found that MSC-Exos effectively inhibited the activation of the nod-like receptor pyrin domain 3 inflammasomes (NLRP3) in macrophages, which in turn decreased the release of proinflammatory factors such as IL-1β and IL-18, thereby effectively alleviating OA [116]. Moreover, MSC-Exos has a significant effect on M1/M2 polarization. The ability of exosomes to alter macrophage polarization can be developed for a variety of medical purposes [117]. Zhang et al. [84] have demonstrated that MSC-Exos can attract M2 macrophages to infiltrate OA joint cavities while reducing M1 macrophage infiltration, downregulating IL-1β and TNF-α expression, and effectively controlling OA progression. Other disease models have also been used to investigate MSC-Exos in driving M1 and M2 polarization and reducing inflammation [118]. Several studies have revealed that MSC-Exos-derived miRNAs play an essential role in regulating macrophage polarization [119, 120]. In addition, the regulation of T and B cells by MSC-Exos is also involved in inflammation. In vitro experiments have demonstrated that MSC-Exos can induce the conversion of T1 helper (Th1) cells to Th2 cells and inhibit the differentiation of T cells to Th17 cells, thus increasing the subpopulation of regulatory T cells (Tregs) [121]. This implies that MSC-Exos can decrease proinflammatory T helper cells Th1 [122] and Th17 [123], while Tregs act as immunomodulators that suppress immune responses and maintain immune tolerance by secreting cytokines such as TGF-β, IL-10, and IL-35, ultimately suppressing inflammation [124]. Studies on an in vivo arthritis model have shown that MSC-Exos effectively inhibits T-cell proliferation while inducing Treg proliferation, resulting in improved inflammatory progression. In contrast, MSC-Exos inhibit the terminal differentiation and maturation of plasma cells, possibly by inhibiting the expression of chemokine receptors on B cells, thereby suppressing their proliferation and reducing their chemotaxis [125]. Although MSC-Exos regulation of T and B cells in OA has yet to be fully understood, macrophage-related studies have demonstrated their important role in immune regulation, providing a new perspective for OA treatment.

## Bioengineering solutions to enhance the innate functions of MSC-Exos toward osteoarthritis therapy

Preclinical animal studies have demonstrated promising responses for the use of MSC-Exos in the treatment of OA. However, the innate function of MSC-Exos is not always therapeutically adequate for disease treatment [126]. To maximize the targeted therapeutic efficacy of MSC-Exos, several bioengineering modification options should be explored to improve the innate function of MSC-Exos [94, 127–130]. Production of exosomes can also be optimized for process optimization, including the possibility of obtaining exosomes or biomimetic exosomes from three-dimensional (3D) culture [131]. In addition, 3D printing technology has gained increasing attention in recent years, and 3D printing-based tissue engineering strategies could be a novel solution to enhance the residence time and function of exosomes. Additionally, priming MSCs with hypoxia, cytokines, and small molecules has improved the therapeutic potential of MSC-exos.

### Priming MSC-derived exosomes with TGF-β1

In particular, the growth factor TGF-β plays an important role in promoting the biological functions of MSC-Exos. Studies have shown that TGF-β1-stimulated MSC-Exos can promote cartilage repair. Alternatively, coculturing TGF-β1 with MSCs can upregulate the expression of miRNA-135b in MSC-Exos, which serves as a potent inhibitor to downregulate Sp1 protein, a class of transcription factors, which is associated with apoptosis. Researchers found that Sp1 can inhibits chondrocyte proliferation by upregulating the mouse collagen α1 (XI) gene [132]. Further investigations revealed that TGF-β1 exosome-treated murine chondrocytes displayed reduced Sp1 expression and enhanced cell viability, indicating that TGF-β1 downregulated Sp1 through MSC-Exo-derived miR-135b to improve the therapeutic outcome in an OA rat model [133]. Similarly, TGF-β1-modified MSC-Exo-derived miR-135b attenuated cartilage damage in OA rats by promoting M2 macrophage polarization [134]. In conclusion, priming MSCs with TGF-β1 can promote the function of MSC-Exos in promoting chondrocyte proliferation. In another study, TGF-β1 combined with BM-MSC-exos ameliorated articular cartilage degeneration, maintained local bone homeostasis, and alleviated pain, effectively mitigating disease progression in OA mice [135]. Therefore, the use of TGF-β1-enhanced exosomes for OA therapy represents a novel promising strategy.

### Priming MSC-derived exosomes with TGF-β3 and KGN

TGF-β3 has been found to be more beneficial than TGF-β1 in stimulating cartilage formation [136]. Research has shown that MSC-Exos can promote the expression of chondrocyte surface markers aggrecan and type II collagen and reduce the expression of catabolic markers such as MMP-13 and ADAMTS5, and inflammatory markers such as iNOS. These effects can be further enhanced when BM-MSCs are preactivated with TGF-β3 [69]. However, TGF-β3 also promotes the expression of type X collagen genes during the late induction phase, leading to hypertrophy and eventually the formation of non-functional fibrocartilage rather than hyaline cartilage [136, 137]. In contrast, a novel small molecule compound KGN has been shown to inhibit TGF-β3-induced hypertrophy [138]. Therefore, priming BM-MSC-Exos with KGN (KGN-BMSC-Exos) was more effective in increasing the expression levels of GAG, COL-II, prg4, and SOX-9 compared with BM-MSC-Exos, suggesting that KGN-BMSC-Exos promote the synthesis of cartilage matrix [139]. Another study showed that costimulating rabbit SF-MSCs with KGN and TGF-β3 in vitro significantly increased the protein expression levels of type II collagen (COL II) and SRY-box 9 (SOX9) and decreased the expression level of type X collagen (COL X). These results demonstrated that the combined application of TGF-β3 and KGN can increase the expression of hyaline-like cartilage markers in vitro and improve the repair effect in vivo [29]. Overall, these results indicate that pretreatment of MSC-Exos with KGN and TGF-β3 represents another promising approach to enhance the therapeutic efficacy of MSC-Exos.

### Gene transfection enhances the therapeutic effect of MSCs-derived exosomes

Achieving cost-efficiency in the production and acquisition of exosomes is crucial due to the time-consuming and expensive nature of the process. To address this issue, several studies have explored the transfection of the *MYC* gene into MSCs to enable large production of exosomes [140]. In one study, MSCs carrying *MYC gene* ensured an unlimited supply of cells, greatly increasing exosome production efficiency and reducing production costs [141]. Transfection of MSCs with a lentiviral vector containing the *MYC* gene has been shown to transform MSCs with delayed senescence and accelerated growth factor secretion, allowing for scale-up production of MSCs without senescence. As a result, transfected MSCs have been reported to secrete significant quantities of exosomes containing therapeutic factors, such as miR-92a-3p [90], lncRNA-KLF3-AS1 [86], miR-140-5p [111], and miR-320c [112], with notable therapeutic effects on OA. However, gene transfection has been controversial and they are still a long way entering into clinic trial. In particular, the *MYC* gene, as a class of protooncogenes, produces the most commonly activated oncoproteins in human cancers. Numerous studies have demonstrated that overexpression of *MYC* gene causes tumorigenesis [142]. Fortunately, in vitro studies have found that expression of *MYC* proteins in exosomes derived from *MYC*-transfected MSCs can not detected, thus the risk of carcinogenicity of the collected exosomes was greatly reduced [141]. Although it is unlikely that *MYC*-transformed MSCs can be used for clinical applications at this time, in vitro use for larger production of exosomes seems to be a strategy that deserves further investigation. New gene editing tools such as CRISPR/ Cas9 can also be used to enhance the function of exosomes, and it is believed that similar gene-edited exosomes will be available as novel way to change the therapy function [143–146].

### Three-dimensional cultures enhance the therapeutic capacities of MSC-derived exosomes

The productivity of exosomes can be continuously and efficiently increased by using the 3D culture technique. Many studies have compared the size, content, function, and production efficiency of exosomes in 2D and 3D cultures. The results clearly and consistently demonstrated that exosomes cultured in 3D have superior productivity and therapeutic effects than those cultured in 2D [147]. In multicellular organisms, cells are highly organized by the extracellular matrix in a 3D manner, while 2D cultures lack the spatial polarization and structures found in vivo, leading to alterations in exosome morphology, contents, and functions [148]. To obtain meaningful results, researchers have prepared 3D scaffolds using cell-free cartilage extracellular matrix to produce more exosomes, which resulted in stronger cartilage repair in a rat model of knee cartilage defect [149]. In addition, collecting exosomes by 3D-culturing MSCs can maintain the MSCs phenotype and produce exosomes with higher therapeutic efficiency [26, 150]. For example, Cao et al. [151] showed that a 3D culture system increased the total yield of MSC-Exos by 19.4-fold, with higher production efficiency and excellent antiinflammatory effects. Therefore, 3D culture technology may be a promising method to improve the secretion of MSC-Exos for the treatment of OA.

### MSC-exosome-laden scaffolds for enhancing osteoarthritis cartilage tissue repair

In recent years, some natural or polymeric biomaterials have gained widespread use in cartilage repair and regeneration. These biomaterial-based scaffolds provide mechanical and 3D structure support, facilitating cell adhesion, migration, and differentiation in vivo [152, 153]. For OA, cartilage defect repair has been a challenging task, and the use of scaffold materials may enable ideal cartilage defect repair outcomes. Scaffold materials can be used as ideal patches to fill in cartilage defects and retain MSC-Exos at the cartilage defect site, ensuring precise and prolonged biological effects that induce cartilage repair and reverse OA progression [154, 155].

Hydrogels are one of the preferred scaffold materials due to their high water content, biocompatibility, swelling behavior, and adjustable 3D network [156]. Liu et al. revealed the superior efficacy of photo-induced imine cross-linked hydrogels as exosome scaffolds in repairing OA. These composite scaffolds have excellent cartilage integration, can bind to natural cartilage matrix, and promote cell deposition at the cartilage defect site. Furthermore, MSC-Exos could be retained long-term with the scaffolds’ support, ultimately promoting cartilage defect repair [157]. In another study, 3D-printed cartilage ECM/gelatin methacrylate (GelMA) scaffolds were used to support exosomes. The scaffolds were evaluated in a rabbit OA model, and the results showed that the ECM/GelMA/exosome scaffolds effectively repaired chondrocyte mitochondrial dysfunction, promoted cartilage cell migration, and differentiated synovial macrophages toward the M2 phenotype [158]. Jiang et al. used cell-free cartilage extracellular matrix (ACECM) scaffold loaded with human umbilical cord Wharton lyophilized cell exosomes (hWJMSC-Exos) and found it enhanced the effect of the ACECM scaffold, demonstrating a superior prochondrogenic effect in a rabbit knee osteochondral defect repair model [159]. Overall, the scaffold materials loaded with MSC-Exos have significant potential for enhancing cartilage repair and improving the inflammatory environment of the joint cavity, providing a potent and predictable therapeutic strategy for OA.

## Conclusions and prospects

Although the immunomodulatory and regenerative properties of MSCs have demonstrated their potential in OA therapies, challenges such as sourcing, culture condition, preservation, and possible host conflicts after transplantation remain. However, the study of the functions of MSC-generated exosomes has revealed their great potential for the treatment of OA. MSC-Exos are considered a key factor for their small size, stability, biological activity, and targeting property in the treatment of OA. To further expand the therapeutic scope of MSC-Exos beyond their inherent functions, engineering approaches can significantly improve the efficacy of clinical OA indications. For example, pretreating MSCs with substances such as TGF-β1, TFG-β3, and KGN and establishing a rational 3D culture microenvironment to promote exosome production and functional enhancement can increase the potency of MSCs. Strategies such as the use of biomaterials can help localize exosome injections directly to the damaged target site for long-lasting effects [160]. Future studies should explore the utilization of large animal models to better mimic OA conditions and clinical dosing regimens. However, before we discuss the clinical translation of exosome therapy for OA, it is necessary to explore the current progress of clinical translation of MSC-derived extracellular vesicles (MSC-EVs), which can give researchers and patients new therapeutic strategies and the direction for future research. EVs are cargo-carrying vesicles, including exosomes, that are released by cells into the extracellular space [161]. In particular, MSC-secreted EVs can show comparable biological effects out of parental cells and exhibit more stability and safety, thus researchers have exploited a lot of efforts for clinical application [162, 163]. According to the registered projects of the clinical trials database, they are about studies on MSC-EVs, with more than 3000 subjects expected to receive MSC-EVs for a variety of diseases, including acute respiratory distress syndrome [164, 165]. These clinical trials have given new potential for the treatment of patients in the corresponding fields, especially exosome-based therapy for OA.

Although we have proposed many of the advantages of exosomes, the current research on exosome therapy for OA is limited to the preclinical animal phase. Two technical obstacles mainly limit the application of exosomes—on the one hand, current technology makes it difficult to obtain exosomes in a simplified and high yield; on the other hand, efforts are still needed to efficiently isolate functional exosomes [166]. In the above discussion, we illustrated examples of exosome yield enhancement through bioengineering, genetic, and 3D technologies, which indicate the direction of future technological breakthroughs. However, all currently used techniques for exosome isolation have corresponding drawbacks, and none of them can be standardized [167]. Even specific exosomes hide numerous problems due to the wrapping of various complex molecules, including the uncontrollability of the effects produced by unknown molecules and the quantification of miRNA, lncRNA, and circRNA in MSC-Exos [168]. In addition, there is still a lack of pharmacokinetics and biodistribution of exosomes in vivo [169, 170]. Undeniably, several organizations, including the International Society for Extracellular Vesicles, are try to develop guidelines for MSC-Exos for the treatment of OA [171]. As work continues, the resolution of issues such as idealized sources of exosome, standardized access, delivery routes, dosing, and dosing frequency is key to bringing exosome therapy for OA into the clinic setting [168].

It is believed that exosomes will play a greater role as the next generation of “mesenchymal stem cell therapy” in treating OA. One day, MSC-Exos for OA will again clinically benefit humanity.

## Data Availability

Data will be made available on request.

## Abbreviations

OA: Osteoarthritis
MSCs: Mesenchymal stem cells
MSC-Exos: Mesenchymal stem cell-derived exosomes
IL: Interleukin
TNF: Tumor necrosis factor
TGF: Transforming growth factor
MMP: Matrix metalloproteinases
JNK: C-Jun N-terminal kinase
KGN: Kartogenin
ncRNAs: Non-coding RNAs
SFB: Synovial fibroblasts
IPFP: Infrapatellar fat pads
ECM: Extracellular matrix
DAMP: Damage-associated molecular patterns
ACI: Autologous chondrocyte implantation
MACI: Matrix-induced autologous chondrocyte implantation
BM-MSCs: Bone marrow-derived MSCs
SF-MSCs: Synovial fluid-derived MSCs
AD-MSCs: Adipose tissue-derived MSCs
PB: Peripheral blood
SDF-1: Stromal derived factor-1
CXCR: Chemokine receptors
BMP: Bone morphogenetic protein
ILVs: Intraluminal vesicles
MVBs: Multivesicular bodies
ESCRT: Endosomal sorting complex required for transport
ALIX: ALG-2-interacting protein X
VPS4: Vacuolar protein sorting-associated protein 4
miRNA: MicroRNA
lncRNA: Long non-coding RNA
RBPs: RNA binding proteins
Th cell: Helper T cell
Sp1: Specificity protein 1
MSC-EVs: MSC derived extracellular vesicles

## References

[1] Loeser RF, Goldring SR, Scanzello CR, Goldring MB (2012). Osteoarthritis: a disease of the joint as an organ. Arthritis Rheum.

[2] Muthu S (2023). Osteoarthritis, an old wine in a new bottle!. World J Orthop.

[3] Quicke JG, Conaghan PG, Corp N, Peat G (2022). Osteoarthritis year in review 2021: epidemiology & therapy. Osteoarthritis Cartilage.

[4] Dobson GP, Letson HL, Grant A, McEwen P, Hazratwala K, Wilkinson M (2018). Defining the osteoarthritis patient: back to the future. Osteoarthritis Cartilage.

[5] de Lange-Brokaar BJ, Ioan-Facsinay A, van Osch GJ, Zuurmond AM, Schoones J, Toes RE (2012). Synovial inflammation, immune cells and their cytokines in osteoarthritis: a review. Osteoarthritis Cartilage.

[6] Man GS, Mologhianu G (2014). Osteoarthritis pathogenesis—a complex process that involves the entire joint. J Med Life.

[7] Chen D, Shen J, Zhao W, Wang T, Han L, Hamilton JL (2017). Osteoarthritis: toward a comprehensive understanding of pathological mechanism. Bone Res.

[8] Hunter DJ, Bierma-Zeinstra S (2019). Osteoarthritis. Lancet (London, England).

[9] Troeberg L, Fushimi K, Scilabra SD, Nakamura H, Dive V, Thøgersen IB (2009). The C-terminal domains of ADAMTS-4 and ADAMTS-5 promote association with N-TIMP-3. Matrix Biol.

[10] Duan L, Liang Y, Xu X, Xiao Y, Wang D (2020). Recent progress on the role of miR-140 in cartilage matrix remodelling and its implications for osteoarthritis treatment. Arthritis Res Ther.

[11] Liang Y, Duan L, Xiong J, Zhu W, Liu Q, Wang D (2016). E2 regulates MMP-13 via targeting miR-140 in IL-1β-induced extracellular matrix degradation in human chondrocytes. Arthritis Res Ther.

[12] Wu Y, Lu X, Shen B, Zeng Y (2019). The therapeutic potential and role of miRNA, lncRNA, and circRNA in osteoarthritis. Curr Gene Ther.

[13] Liang Y, Xu X, Li X, Xiong J, Li B, Duan L (2020). Chondrocyte-targeted MicroRNA delivery by engineered exosomes toward a cell-free osteoarthritis therapy. ACS Appl Mater Interfaces.

[14] Kim HA, Blanco FJ (2007). Cell death and apoptosis in osteoarthritic cartilage. Curr Drug Targets.

[15] Miwa M, Saura R, Hirata S, Hayashi Y, Mizuno K, Itoh H (2000). Induction of apoptosis in bovine articular chondrocyte by prostaglandin E(2) through cAMP-dependent pathway. Osteoarthritis Cartilage.

[16] Sharma AR, Jagga S, Lee SS, Nam JS (2013). Interplay between cartilage and subchondral bone contributing to pathogenesis of osteoarthritis. Int J Mol Sci.

[17] Mathiessen A, Conaghan PG (2017). Synovitis in osteoarthritis: current understanding with therapeutic implications. Arthritis Res Ther.

[18] Clockaerts S, Bastiaansen-Jenniskens YM, Runhaar J, Van Osch GJ, Van Offel JF, Verhaar JA (2010). The infrapatellar fat pad should be considered as an active osteoarthritic joint tissue: a narrative review. Osteoarthritis Cartilage.

[19] Khan M, Osman K, Green G, Haddad FS (2016). The epidemiology of failure in total knee arthroplasty: avoiding your next revision. Bone Joint J.

[20] Toh WS, Lai RC, Hui JHP, Lim SK (2017). MSC exosome as a cell-free MSC therapy for cartilage regeneration: implications for osteoarthritis treatment. Semin Cell Dev Biol.

[21] Pittenger MF, Mackay AM, Beck SC, Jaiswal RK, Douglas R, Mosca JD (1999). Multilineage potential of adult human mesenchymal stem cells. Science.

[22] Al-Azab M, Safi M, Idiiatullina E, Al-Shaebi F, Zaky MY (2022). Aging of mesenchymal stem cell: machinery, markers, and strategies of fighting. Cell Mol Biol Lett.

[23] Lopa S, Colombini A, Moretti M, de Girolamo L (2019). Injective mesenchymal stem cell-based treatments for knee osteoarthritis: from mechanisms of action to current clinical evidences. Knee Surg Sports Traumatol Arthrosc.

[24] Jones EA, Crawford A, English A, Henshaw K, Mundy J, Corscadden D (2008). Synovial fluid mesenchymal stem cells in health and early osteoarthritis: detection and functional evaluation at the single-cell level. Arthritis Rheum.

[25] Neybecker P, Henrionnet C, Pape E, Mainard D, Galois L, Loeuille D (2018). In vitro and in vivo potentialities for cartilage repair from human advanced knee osteoarthritis synovial fluid-derived mesenchymal stem cells. Stem Cell Res Ther.

[26] Duan L, Li X, Xu X, Xu L, Wang D, Ouyang K, et al. Large-scale preparation of synovial fluid mesenchymal stem cell-derived exosomes by 3D bioreactor culture. J Visualized Exp JoVE. 2022(185). 10.3791/62221 35969064

[27] Jia Z, Liang Y, Li X, Xu X, Xiong J, Wang D, et al. Magnetic-activated cell sorting strategies to isolate and purify synovial fluid-derived mesenchymal stem cells from a rabbit model. J Visualized Exp JoVE. 2018(138). 10.3791/57466 PMC6126689 30148486

[28] Xu X, Liang Y, Li X, Ouyang K, Wang M, Cao T (2021). Exosome-mediated delivery of kartogenin for chondrogenesis of synovial fluid-derived mesenchymal stem cells and cartilage regeneration. Biomaterials.

[29] Jia Z, Wang S, Liang Y, Liu Q (2019). Combination of kartogenin and transforming growth factor-β3 supports synovial fluid-derived mesenchymal stem cell-based cartilage regeneration. Am J Transl Res.

[30] Jia Z, Liu Q, Liang Y, Li X, Xu X, Ouyang K (2018). Repair of articular cartilage defects with intra-articular injection of autologous rabbit synovial fluid-derived mesenchymal stem cells. J Transl Med.

[31] Xu X, Xu L, Xia J, Wen C, Liang Y, Zhang Y (2023). Harnessing knee joint resident mesenchymal stem cells in cartilage tissue engineering. Acta Biomaterialia.

[32] Muthu S, Patil SC, Jeyaraman N, Jeyaraman M, Gangadaran P, Rajendran RL (2023). Comparative effectiveness of adipose-derived mesenchymal stromal cells in the management of knee osteoarthritis: a meta-analysis. World J Orthop.

[33] Uzieliene I, Urbonaite G, Tachtamisevaite Z, Mobasheri A, Bernotiene E (2018). The potential of menstrual blood-derived mesenchymal stem cells for cartilage repair and regeneration: novel aspects. Stem Cells Int.

[34] Wang SJ, Jiang D, Zhang ZZ, Huang AB, Qi YS, Wang HJ (2016). Chondrogenic potential of peripheral blood derived mesenchymal stem cells seeded on demineralized cancellous bone scaffolds. Sci Rep.

[35] Khalifeh Soltani S, Forogh B, Ahmadbeigi N, Hadizadeh Kharazi H, Fallahzadeh K, Kashani L (2019). Safety and efficacy of allogenic placental mesenchymal stem cells for treating knee osteoarthritis: a pilot study. Cytotherapy.

[36] Gomez-Leduc T, Hervieu M, Legendre F, Bouyoucef M, Gruchy N, Poulain L (2016). Chondrogenic commitment of human umbilical cord blood-derived mesenchymal stem cells in collagen matrices for cartilage engineering. Sci Rep.

[37] Miranda-Sayago JM, Fernandez-Arcas N, Benito C, Reyes-Engel A, Carrera J, Alonso A (2011). Lifespan of human amniotic fluid-derived multipotent mesenchymal stromal cells. Cytotherapy.

[38] Ahmed AS, Sheng MH, Wasnik S, Baylink DJ, Lau KW (2017). Effect of aging on stem cells. World J Exp Med.

[39] Zhou T, Yuan Z, Weng J, Pei D, Du X, He C (2021). Challenges and advances in clinical applications of mesenchymal stromal cells. J Hematol Oncol.

[40] Lamo-Espinosa JM, Blanco JF, Sanchez M, Moreno V, Granero-Molto F, Sanchez-Guijo F (2020). Phase II multicenter randomized controlled clinical trial on the efficacy of intra-articular injection of autologous bone marrow mesenchymal stem cells with platelet rich plasma for the treatment of knee osteoarthritis. J Transl Med.

[41] Davatchi F, Abdollahi BS, Mohyeddin M, Shahram F, Nikbin B (2011). Mesenchymal stem cell therapy for knee osteoarthritis. Preliminary report of four patients. Int J Rheum Dis.

[42] Emadedin M, Labibzadeh N, Liastani MG, Karimi A, Jaroughi N, Bolurieh T (2018). Intra-articular implantation of autologous bone marrow-derived mesenchymal stromal cells to treat knee osteoarthritis: a randomized, triple-blind, placebo-controlled phase 1/2 clinical trial. Cytotherapy.

[43] Koh YG, Choi YJ (2012). Infrapatellar fat pad-derived mesenchymal stem cell therapy for knee osteoarthritis. Knee.

[44] Lamo-Espinosa JM, Mora G, Blanco JF, Granero-Molto F, Nunez-Cordoba JM, Sanchez-Echenique C (2016). Intra-articular injection of two different doses of autologous bone marrow mesenchymal stem cells versus hyaluronic acid in the treatment of knee osteoarthritis: multicenter randomized controlled clinical trial (phase I/II). J Transl Med.

[45] Matas J, Orrego M, Amenabar D, Infante C, Tapia-Limonchi R, Cadiz MI (2019). Umbilical cord-derived mesenchymal stromal cells (MSCs) for knee osteoarthritis: repeated MSC dosing is superior to a single MSC dose and to hyaluronic acid in a controlled randomized phase I/II trial. Stem Cells Transl Med.

[46] Soler R, Orozco L, Munar A, Huguet M, Lopez R, Vives J (2016). Final results of a phase I-II trial using ex vivo expanded autologous Mesenchymal Stromal Cells for the treatment of osteoarthritis of the knee confirming safety and suggesting cartilage regeneration. Knee.

[47] Huang J, Huang Z, Liang Y, Yuan W, Bian L, Duan L (2021). 3D printed gelatin/hydroxyapatite scaffolds for stem cell chondrogenic differentiation and articular cartilage repair. Biomater Sci.

[48] Chahla J, Piuzzi NS, Mitchell JJ, Dean CS, Pascual-Garrido C, LaPrade RF (2016). Intra-articular cellular therapy for osteoarthritis and focal cartilage defects of the knee: a systematic review of the literature and study quality analysis. J Bone Joint Surg Am.

[49] Ding DC, Shyu WC, Lin SZ (2011). Mesenchymal stem cells. Cell Transplant.

[50] Fu X, Liu G, Halim A, Ju Y, Luo Q, Song AG (2019). Mesenchymal stem cell migration and tissue repair. Cells.

[51] Liesveld JL, Sharma N, Aljitawi OS (2020). Stem cell homing: from physiology to therapeutics. Stem Cells.

[52] He J, Jiang B, Dai Y, Hao J, Zhou Z, Tian Z (2013). Regulation of the osteoblastic and chondrocytic differentiation of stem cells by the extracellular matrix and subsequent bone formation modes. Biomaterials.

[53] Goldring MB, Tsuchimochi K, Ijiri K (2006). The control of chondrogenesis. J Cell Biochem.

[54] Caron MM, Emans PJ, Cremers A, Surtel DA, Coolsen MM, van Rhijn LW (2013). Hypertrophic differentiation during chondrogenic differentiation of progenitor cells is stimulated by BMP-2 but suppressed by BMP-7. Osteoarthritis Cartilage.

[55] Zavatti M, Beretti F, Casciaro F, Bertucci E, Maraldi T (2020). Comparison of the therapeutic effect of amniotic fluid stem cells and their exosomes on monoiodoacetate-induced animal model of osteoarthritis. BioFactors.

[56] Andaloussi SEL, Mager I, Breakefield XO, Wood MJ (2013). Extracellular vesicles: biology and emerging therapeutic opportunities. Nat Rev Drug Discov.

[57] van Niel G, D'Angelo G, Raposo G (2018). Shedding light on the cell biology of extracellular vesicles. Nat Rev Mol Cell Biol.

[58] Hurley JH (2015). ESCRTs are everywhere. EMBO J.

[59] Colombo M, Moita C, van Niel G, Kowal J, Vigneron J, Benaroch P (2013). Analysis of ESCRT functions in exosome biogenesis, composition and secretion highlights the heterogeneity of extracellular vesicles. J Cell Sci.

[60] Cheng L, Hill AF (2022). Therapeutically harnessing extracellular vesicles. Nat Rev Drug Discov.

[61] Barile L, Vassalli G (2017). Exosomes: therapy delivery tools and biomarkers of diseases. Pharmacol Ther.

[62] Henne WM, Buchkovich NJ, Emr SD (2011). The ESCRT pathway. Dev Cell.

[63] Hurley JH, Hanson PI (2010). Membrane budding and scission by the ESCRT machinery: it’s all in the neck. Nat Rev Mol Cell Biol.

[64] Verderio C, Gabrielli M, Giussani P (2018). Role of sphingolipids in the biogenesis and biological activity of extracellular vesicles. J Lipid Res.

[65] van Niel G, Charrin S, Simoes S, Romao M, Rochin L, Saftig P (2011). The tetraspanin CD63 regulates ESCRT-independent and -dependent endosomal sorting during melanogenesis. Dev Cell.

[66] Liang Y, Duan L, Lu J, Xia J (2021). Engineering exosomes for targeted drug delivery. Theranostics.

[67] Ostrowski M, Carmo NB, Krumeich S, Fanget I, Raposo G, Savina A (2010). Rab27a and Rab27b control different steps of the exosome secretion pathway. Nat Cell Biol.

[68] Jeppesen DK, Fenix AM, Franklin JL, Higginbotham JN, Zhang Q, Zimmerman LJ (2019). Reassessment of exosome composition. Cell.

[69] Cosenza S, Ruiz M, Toupet K, Jorgensen C, Noël D (2017). Mesenchymal stem cells derived exosomes and microparticles protect cartilage and bone from degradation in osteoarthritis. Sci Rep.

[70] Simons M, Raposo G (2009). Exosomes–vesicular carriers for intercellular communication. Curr Opin Cell Biol.

[71] Di Liegro CM, Schiera G, Di Liegro I (2014). Regulation of mRNA transport, localization and translation in the nervous system of mammals (Review). Int J Mol Med.

[72] Eliscovich C, Buxbaum AR, Katz ZB, Singer RH (2013). mRNA on the move: the road to its biological destiny. J Biol Chem.

[73] Villarroya-Beltri C, Gutierrez-Vazquez C, Sanchez-Cabo F, Perez-Hernandez D, Vazquez J, Martin-Cofreces N (2013). Sumoylated hnRNPA2B1 controls the sorting of miRNAs into exosomes through binding to specific motifs. Nat Commun.

[74] Koppers-Lalic D, Hackenberg M, Bijnsdorp IV, van Eijndhoven MAJ, Sadek P, Sie D (2014). Nontemplated nucleotide additions distinguish the small RNA composition in cells from exosomes. Cell Rep.

[75] Wei Z, Batagov AO, Schinelli S, Wang J, Wang Y, El Fatimy R (2017). Coding and noncoding landscape of extracellular RNA released by human glioma stem cells. Nat Commun.

[76] Kosaka N, Iguchi H, Yoshioka Y, Takeshita F, Matsuki Y, Ochiya T (2010). Secretory mechanisms and intercellular transfer of microRNAs in living cells. J Biol Chem.

[77] Mateescu B, Kowal EJ, van Balkom BW, Bartel S, Bhattacharyya SN, Buzas EI (2017). Obstacles and opportunities in the functional analysis of extracellular vesicle RNA—an ISEV position paper. J Extracell Vesicles.

[78] Takahashi A, Okada R, Nagao K, Kawamata Y, Hanyu A, Yoshimoto S (2017). Exosomes maintain cellular homeostasis by excreting harmful DNA from cells. Nat Commun.

[79] Yokoi A, Villar-Prados A, Oliphint PA, Zhang J, Song X, De Hoff P (2019). Mechanisms of nuclear content loading to exosomes. Sci Adv.

[80] Hitomi K, Okada R, Loo TM, Miyata K, Nakamura AJ, Takahashi A (2020). DNA damage regulates senescence-associated extracellular vesicle release via the ceramide pathway to prevent excessive inflammatory responses. Int J Mol Sci.

[81] Fang W, Vangsness CT, Jr. Implications of anti-inflammatory nature of exosomes in knee arthritis. Cartilage. 2020:1947603520904766. 10.1177/1947603520904766 PMC8804870 32052641

[82] Mianehsaz E, Mirzaei HR, Mahjoubin-Tehran M, Rezaee A, Sahebnasagh R, Pourhanifeh MH (2019). Mesenchymal stem cell-derived exosomes: a new therapeutic approach to osteoarthritis?. Stem Cell Res Ther.

[83] Xie F, Liu YL, Chen XY, Li Q, Zhong J, Dai BY (2020). Role of MicroRNA, LncRNA, and exosomes in the progression of osteoarthritis: a review of recent literature. Orthop Surg.

[84] Zhang S, Chuah SJ, Lai RC, Hui JHP, Lim SK, Toh WS (2018). MSC exosomes mediate cartilage repair by enhancing proliferation, attenuating apoptosis and modulating immune reactivity. Biomaterials.

[85] Qiu B, Xu X, Yi P, Hao Y (2020). Curcumin reinforces MSC-derived exosomes in attenuating osteoarthritis via modulating the miR-124/NF-kB and miR-143/ROCK1/TLR9 signalling pathways. J Cell Mol Med.

[86] Liu Y, Lin L, Zou R, Wen C, Wang Z, Lin F (2018). MSC-derived exosomes promote proliferation and inhibit apoptosis of chondrocytes via lncRNA-KLF3-AS1/miR-206/GIT1 axis in osteoarthritis. Cell Cycle.

[87] Fu Y, Cui S, Zhou Y, Qiu L (2023). Dental pulp stem cell-derived exosomes alleviate mice knee osteoarthritis by inhibiting TRPV4-mediated osteoclast activation. Int J Mol Sci.

[88] Jin Y, Xu M, Zhu H, Dong C, Ji J, Liu Y (2021). Therapeutic effects of bone marrow mesenchymal stem cells-derived exosomes on osteoarthritis. J Cell Mol Med.

[89] Liao Q, Li BJ, Li Y, Xiao Y, Zeng H, Liu JM (2021). Low-intensity pulsed ultrasound promotes osteoarthritic cartilage regeneration by BMSC-derived exosomes via modulating the NF-kappaB signaling pathway. Int Immunopharmacol.

[90] Mao G, Zhang Z, Hu S, Zhang Z, Chang Z, Huang Z (2018). Exosomes derived from miR-92a-3p-overexpressing human mesenchymal stem cells enhance chondrogenesis and suppress cartilage degradation via targeting WNT5A. Stem Cell Res Ther.

[91] Wang Y, Yu D, Liu Z, Zhou F, Dai J, Wu B (2017). Exosomes from embryonic mesenchymal stem cells alleviate osteoarthritis through balancing synthesis and degradation of cartilage extracellular matrix. Stem Cell Res Ther.

[92] Wu J, Kuang L, Chen C, Yang J, Zeng WN, Li T (2019). miR-100-5p-abundant exosomes derived from infrapatellar fat pad MSCs protect articular cartilage and ameliorate gait abnormalities via inhibition of mTOR in osteoarthritis. Biomaterials.

[93] Zhang S, Teo KYW, Chuah SJ, Lai RC, Lim SK, Toh WS (2019). MSC exosomes alleviate temporomandibular joint osteoarthritis by attenuating inflammation and restoring matrix homeostasis. Biomaterials.

[94] Li DF, Liu QS, Yang MF, Xu HM, Zhu MZ, Zhang Y (2023). Nanomaterials for mRNA-based therapeutics: challenges and opportunities. Bioeng Transl Med.

[95] Bartel DP (2009). MicroRNAs: target recognition and regulatory functions. Cell.

[96] Mo YY (2012). MicroRNA regulatory networks and human disease. Cell Mol Life Sci.

[97] Gebert LFR, MacRae IJ (2019). Regulation of microRNA function in animals. Nat Rev Mol Cell Biol.

[98] Mihanfar A, Shakouri SK, Khadem-Ansari MH, Fattahi A, Latifi Z, Nejabati HR (2020). Exosomal miRNAs in osteoarthritis. Mol Biol Rep.

[99] Ballantyne MD, McDonald RA, Baker AH (2016). lncRNA/MicroRNA interactions in the vasculature. Clin Pharmacol Ther.

[100] Liu Y, Zou R, Wang Z, Wen C, Zhang F, Lin F (2018). Exosomal KLF3-AS1 from hMSCs promoted cartilage repair and chondrocyte proliferation in osteoarthritis. Biochem J.

[101] Patop IL, Kadener S (2018). circRNAs in Cancer. Curr Opin Genet Dev.

[102] Zhu C, Shen K, Zhou W, Wu H, Lu Y (2021). Exosome-mediated circ_0001846 participates in IL-1beta-induced chondrocyte cell damage by miR-149-5p-dependent regulation of WNT5B. Clin Immunol.

[103] Guo Z, Wang H, Zhao F, Liu M, Wang F, Kang M (2021). Exosomal circ-BRWD1 contributes to osteoarthritis development through the modulation of miR-1277/TRAF6 axis. Arthritis Res Ther.

[104] Iliopoulos D, Malizos KN, Oikonomou P, Tsezou A (2008). Integrative microRNA and proteomic approaches identify novel osteoarthritis genes and their collaborative metabolic and inflammatory networks. PLoS ONE.

[105] Ham O, Song BW, Lee SY, Choi E, Cha MJ, Lee CY (2012). The role of microRNA-23b in the differentiation of MSC into chondrocyte by targeting protein kinase A signaling. Biomaterials.

[106] Ning G, Liu X, Dai M, Meng A, Wang Q (2013). MicroRNA-92a upholds Bmp signaling by targeting noggin3 during pharyngeal cartilage formation. Dev Cell.

[107] Mao G, Hu S, Zhang Z, Wu P, Zhao X, Lin R (2018). Exosomal miR-95-5p regulates chondrogenesis and cartilage degradation via histone deacetylase 2/8. J Cell Mol Med.

[108] Matsukawa T, Sakai T, Yonezawa T, Hiraiwa H, Hamada T, Nakashima M (2013). MicroRNA-125b regulates the expression of aggrecanase-1 (ADAMTS-4) in human osteoarthritic chondrocytes. Arthritis Res Ther.

[109] Meng F, Zhang Z, Chen W, Huang G, He A, Hou C (2016). MicroRNA-320 regulates matrix metalloproteinase-13 expression in chondrogenesis and interleukin-1beta-induced chondrocyte responses. Osteoarthritis Cartilage.

[110] Karlsen TA, Jakobsen RB, Mikkelsen TS, Brinchmann JE (2014). microRNA-140 targets RALA and regulates chondrogenic differentiation of human mesenchymal stem cells by translational enhancement of SOX9 and ACAN. Stem Cells Dev.

[111] Tao SC, Yuan T, Zhang YL, Yin WJ, Guo SC, Zhang CQ (2017). Exosomes derived from miR-140-5p-overexpressing human synovial mesenchymal stem cells enhance cartilage tissue regeneration and prevent osteoarthritis of the knee in a rat model. Theranostics.

[112] Sun H, Hu S, Zhang Z, Lun J, Liao W, Zhang Z (2019). Expression of exosomal microRNAs during chondrogenic differentiation of human bone mesenchymal stem cells. J Cell Biochem.

[113] Zhao L, Chen S, Yang P, Cao H, Li L (2019). The role of mesenchymal stem cells in hematopoietic stem cell transplantation: prevention and treatment of graft-versus-host disease. Stem Cell Res Ther.

[114] de Castro LL, Lopes-Pacheco M, Weiss DJ, Cruz FF, Rocco PRM (2019). Current understanding of the immunosuppressive properties of mesenchymal stromal cells. J Mol Med (Berl).

[115] Guillamat-Prats R, Camprubi-Rimblas M, Bringue J, Tantinya N, Artigas A (2017). Cell therapy for the treatment of sepsis and acute respiratory distress syndrome. Ann Transl Med.

[116] Zhou H, Shen X, Yan C, Xiong W, Ma Z, Tan Z (2022). Extracellular vesicles derived from human umbilical cord mesenchymal stem cells alleviate osteoarthritis of the knee in mice model by interacting with METTL3 to reduce m6A of NLRP3 in macrophage. Stem Cell Res Ther.

[117] Gharavi AT, Hanjani NA, Movahed E, Doroudian M (2022). The role of macrophage subtypes and exosomes in immunomodulation. Cell Mol Biol Lett.

[118] Sun G, Li G, Li D, Huang W, Zhang R, Zhang H (2018). hucMSC derived exosomes promote functional recovery in spinal cord injury mice via attenuating inflammation. Mater Sci Eng C Mater Biol Appl.

[119] Song Y, Dou H, Li X, Zhao X, Li Y, Liu D (2017). Exosomal miR-146a contributes to the enhanced therapeutic efficacy of interleukin-1beta-primed mesenchymal stem cells against sepsis. Stem Cells.

[120] Jiang M, Wang H, Jin M, Yang X, Ji H, Jiang Y (2018). Exosomes from MiR-30d-5p-ADSCs reverse acute ischemic stroke-induced, autophagy-mediated brain injury by promoting M2 microglial/macrophage polarization. Cell Physiol Biochem.

[121] Chen W, Huang Y, Han J, Yu L, Li Y, Lu Z (2016). Immunomodulatory effects of mesenchymal stromal cells-derived exosome. Immunol Res.

[122] Li YS, Luo W, Zhu SA, Lei GH (2017). T cells in osteoarthritis: alterations and beyond. Front Immunol.

[123] Bommireddy R, Doetschman T (2007). TGFbeta1 and Treg cells: alliance for tolerance. Trends Mol Med.

[124] Hori S, Nomura T, Sakaguchi S (2003). Control of regulatory T cell development by the transcription factor Foxp3. Science.

[125] Khare D, Or R, Resnick I, Barkatz C, Almogi-Hazan O, Avni B (2018). Mesenchymal stromal cell-derived exosomes affect mRNA expression and function of B-lymphocytes. Front Immunol.

[126] Bader P, Kuçi Z, Bakhtiar S, Basu O, Bug G, Dennis M (2018). Effective treatment of steroid and therapy-refractory acute graft-versus-host disease with a novel mesenchymal stromal cell product (MSC-FFM). Bone Marrow Transplant.

[127] Liang Y, Iqbal Z, Lu J, Wang J, Zhang H, Chen X (2023). Cell-derived nanovesicle-mediated drug delivery to the brain: principles and strategies for vesicle engineering. Mol Ther.

[128] Xu L, Xu X, Liang Y, Wen C, Ouyang K, Huang J (2023). Osteoclast-targeted delivery of anti-miRNA oligonucleotides by red blood cell extracellular vesicles. J Controlled Release.

[129] Liang Y, Xu X, Xu L, Iqbal Z, Ouyang K, Zhang H (2022). Chondrocyte-specific genomic editing enabled by hybrid exosomes for osteoarthritis treatment. Theranostics.

[130] Duan L, Xu L, Xu X, Qin Z, Zhou X, Xiao Y (2021). Exosome-mediated delivery of gene vectors for gene therapy. Nanoscale.

[131] Xu X, Xu L, Wen C, Xia J, Zhang Y, Liang Y (2023). Programming assembly of biomimetic exosomes: an emerging theranostic nanomedicine platform. Materials Today Bio.

[132] Watanabe K, Hida M, Sasaki T, Yano H, Kawano K, Yoshioka H (2016). Sp1 upregulates the proximal promoter activity of the mouse collagen alpha1(XI) gene (Col11a1) in chondrocytes. In Vitro Cell Dev Biol Anim.

[133] Wang R, Xu B, Xu H (2018). TGF-beta1 promoted chondrocyte proliferation by regulating Sp1 through MSC-exosomes derived miR-135b. Cell Cycle.

[134] Wang R, Xu B (2021). TGF-beta1-modified MSC-derived exosomal miR-135b attenuates cartilage injury via promoting M2 synovial macrophage polarization by targeting MAPK6. Cell Tissue Res.

[135] Wang R, Xu B (2022). TGFbeta1-modified MSC-derived exosome attenuates osteoarthritis by inhibiting PDGF-BB secretion and H-type vessel activity in the subchondral bone. Acta Histochem.

[136] Mueller MB, Fischer M, Zellner J, Berner A, Dienstknecht T, Prantl L (2010). Hypertrophy in mesenchymal stem cell chondrogenesis: effect of TGF-beta isoforms and chondrogenic conditioning. Cells Tissues Organs.

[137] Barry F, Boynton RE, Liu B, Murphy JM (2001). Chondrogenic differentiation of mesenchymal stem cells from bone marrow: differentiation-dependent gene expression of matrix components. Exp Cell Res.

[138] Johnson K, Zhu S, Tremblay MS, Payette JN, Wang J, Bouchez LC (2012). A stem cell-based approach to cartilage repair. Science.

[139] Liu C, Li Y, Yang Z, Zhou Z, Lou Z, Zhang Q (2020). Kartogenin enhances the therapeutic effect of bone marrow mesenchymal stem cells derived exosomes in cartilage repair. Nanomedicine (Lond).

[140] Chang YH, Wu KC, Harn HJ, Lin SZ, Ding DC (2018). Exosomes and stem cells in degenerative disease diagnosis and therapy. Cell Transplant.

[141] Chen TS, Arslan F, Yin Y, Tan SS, Lai RC, Choo AB (2011). Enabling a robust scalable manufacturing process for therapeutic exosomes through oncogenic immortalization of human ESC-derived MSCs. J Transl Med.

[142] Dhanasekaran R, Deutzmann A, Mahauad-Fernandez WD, Hansen AS, Gouw AM, Felsher DW (2022). The MYC oncogene—the grand orchestrator of cancer growth and immune evasion. Nat Rev Clin Oncol.

[143] Iqbal Z, Rehman K, Xia J, Shabbir M, Zaman M, Liang Y (2023). Biomaterial-assisted targeted and controlled delivery of CRISPR/Cas9 for precise gene editing. Biomaterials Sci.

[144] Liang Y, Iqbal Z, Wang J, Xu L, Xu X, Ouyang K (2022). Cell-derived extracellular vesicles for CRISPR/Cas9 delivery: engineering strategies for cargo packaging and loading. Biomaterials Sci.

[145] Duan L, Ouyang K, Wang J, Xu L, Xu X, Wen C (2021). Exosomes as targeted delivery platform of CRISPR/Cas9 for therapeutic genome editing. Chembiochem Eur J Chem Biol.

[146] Duan L, Ouyang K, Xu X, Xu L, Wen C, Zhou X (2021). Nanoparticle delivery of CRISPR/Cas9 for genome editing. Front Genet.

[147] Zhou QF, Cai YZ, Lin XJ (2020). The dual character of exosomes in osteoarthritis: antagonists and therapeutic agents. Acta Biomater.

[148] Lucchetti D, Calapa F, Palmieri V, Fanali C, Carbone F, Papa A (2017). Differentiation affects the release of exosomes from colon cancer cells and their ability to modulate the behavior of recipient cells. Am J Pathol.

[149] Yan Z, Yin H, Wu J, Tian G, Li M, Liao Z (2023). Engineering exosomes by three-dimensional porous scaffold culture of human umbilical cord mesenchymal stem cells promote osteochondral repair. Mater Today Bio.

[150] Han M, Yang H, Lu X, Li Y, Liu Z, Li F (2022). Three-dimensional-cultured MSC-derived exosome-hydrogel hybrid microneedle array patch for spinal cord repair. Nano Lett.

[151] Cao J, Wang B, Tang T, Lv L, Ding Z, Li Z (2020). Three-dimensional culture of MSCs produces exosomes with improved yield and enhanced therapeutic efficacy for cisplatin-induced acute kidney injury. Stem Cell Res Ther.

[152] Jafari M, Paknejad Z, Rad MR, Motamedian SR, Eghbal MJ, Nadjmi N (2017). Polymeric scaffolds in tissue engineering: a literature review. J Biomed Mater Res B Appl Biomater.

[153] Huang J, Xiong J, Yang L, Zhang J, Sun S, Liang Y (2021). Cell-free exosome-laden scaffolds for tissue repair. Nanoscale.

[154] Vilela CA, Correia C, Oliveira JM, Sousa RA, Espregueira-Mendes J, Reis RL (2015). Cartilage repair using hydrogels: a critical review of in vivo experimental designs. ACS Biomater Sci Eng.

[155] Spiller KL, Maher SA, Lowman AM (2011). Hydrogels for the repair of articular cartilage defects. Tissue Eng Part B Rev.

[156] Vega SL, Kwon MY, Burdick JA (2017). Recent advances in hydrogels for cartilage tissue engineering. Eur Cell Mater.

[157] Liu X, Yang Y, Li Y, Niu X, Zhao B, Wang Y (2017). Integration of stem cell-derived exosomes with in situ hydrogel glue as a promising tissue patch for articular cartilage regeneration. Nanoscale.

[158] Chen P, Zheng L, Wang Y, Tao M, Xie Z, Xia C (2019). Desktop-stereolithography 3D printing of a radially oriented extracellular matrix/mesenchymal stem cell exosome bioink for osteochondral defect regeneration. Theranostics.

[159] Jiang S, Tian G, Yang Z, Gao X, Wang F, Li J (2021). Enhancement of acellular cartilage matrix scaffold by Wharton’s jelly mesenchymal stem cell-derived exosomes to promote osteochondral regeneration. Bioact Mater.

[160] Duan L, Xu X, Xu L, Chen H, Li X, Alahdal M (2021). Exosome-mediated drug delivery for cell-free therapy of osteoarthritis. Curr Med Chem.

[161] Couch Y, Buzas EI, Di Vizio D, Gho YS, Harrison P, Hill AF (2021). A brief history of nearly EV-erything—the rise and rise of extracellular vesicles. J Extracell Vesicles.

[162] Courageux Y, Monguio-Tortajada M, Prat-Vidal C, Bayes-Genis A, Roura S (2022). Clinical translation of mesenchymal stromal cell extracellular vesicles: considerations on scientific rationale and production requisites. J Cell Mol Med.

[163] Tian CM, Yang MF, Xu HM, Zhu MZ, Zhang Y, Yao J (2023). Mesenchymal stem cell-derived exosomes: novel therapeutic approach for inflammatory bowel diseases. Stem Cells Int.

[164] Duong A, Parmar G, Kirkham AM, Burger D, Allan DS (2023). Registered clinical trials investigating treatment with cell-derived extracellular vesicles: a scoping review. Cytotherapy.

[165] Tian CM, Zhang Y, Yang MF, Xu HM, Zhu MZ, Yao J (2023). Stem cell therapy in inflammatory bowel disease: a review of achievements and challenges. J Inflamm Res.

[166] Yang D, Zhang W, Zhang H, Zhang F, Chen L, Ma L (2020). Progress, opportunity, and perspective on exosome isolation—efforts for efficient exosome-based theranostics. Theranostics.

[167] He C, Zheng S, Luo Y, Wang B (2018). Exosome theranostics: biology and translational medicine. Theranostics.

[168] Jeyaraman M, Muthu S, Shehabaz S, Jeyaraman N, Rajendran RL, Hong CM (2022). Current understanding of MSC-derived exosomes in the management of knee osteoarthritis. Exp Cell Res.

[169] Taghiyar L, Jahangir S, Khozaei Ravari M, Shamekhi MA, Eslaminejad MB (2021). Cartilage repair by mesenchymal stem cell-derived exosomes: preclinical and clinical trial update and perspectives. Adv Exp Med Biol.

[170] Liu Q, Huang J, Xia J, Liang Y, Li G (2022). Tracking tools of extracellular vesicles for biomedical research. Front Bioeng Biotechnol.

[171] Jeyaraman M, Muthu S, Gulati A, Jeyaraman N, Prajwal GS, Jain R (2021). Mesenchymal stem cell-derived exosomes: a potential therapeutic avenue in knee osteoarthritis. Cartilage.

